# Coronary artery disease in adults with congenital heart disease

**DOI:** 10.1016/j.ijcchd.2023.100466

**Published:** 2023-06-29

**Authors:** Salvatore De Rosa, Jolanda Sabatino, Giovanni Di Salvo, Daniele Torella, Carlo Di Mario

**Affiliations:** a Department of Medical and Surgical Sciences, Magna Graecia University, Catanzaro, Italy; b Division of Paediatric Cardiology, Department of Women's and Children's Health, University Hospital Padua, Padua, Italy; c Pediatric Research Institute (IRP) "Città della Speranza", Padua, Italy; d Department of Experimental and Clinical Medicine, Magna Graecia University, Catanzaro, Italy; e Division of Structural Interventional Cardiology, Department of Clinical & Experimental Medicine, Careggi University Hospital, Florence, Italy

**Keywords:** Congenital heart disease, Coronary artery disease, Grown-up congenital heart disease

## Abstract

The increasing population of adult patients with congenital heart disease (ACHD) is at risk of developing coronary artery disease (CAD) and other atherosclerotic cardiovascular diseases due to exposure to cardiovascular risk factors. The impact of this exposure is growing larger as life expectancy of these subjects increases with the progressive improvement in management of congenital heart disease. Studies have shown that ACHD patients have a higher risk for CAD than their non-ACHD matches, highlighting the need for awareness and prevention efforts among congenital heart disease specialists and non-ACHD cardiologists. At the same time, ACHD patients with CAD often present specific characteristics all practicing cardiologists should be aware of. While further research is needed to fully understand the mechanisms underlying the higher CAD risk in this population, this article summarizes key evidence on CAD in ACHD and emphasizes on one hand the importance of early screening and management of known cardiovascular risk factors in ACHD patients, particularly those who are younger, female, or have more complex/severe CHD. On the other hand, it calls for a broader knowledge of ACHD risk for CAD and its peculiarities among all cardiologists.

## 1 Introduction

About 1% of all newborns are diagnosed with a congenital heart defect (CHD), making it the most common major birth anomaly [1]. As major advances in early diagnosis, medical and surgical management allow more and more children with CHD to grow up and get old, the population of adult patients with congenital heart disease (ACHD) is progressively increasing [[2], [3], [4]]. These adult patients with CHD are exposed to cardiovascular risk factors as their non-ACHD age-matches and might potentially develop coronary artery disease (CAD) and other atherosclerotic cardiovascular diseases, such as cerebrovascular disease and heart failure [5,6]. Increasing evidence suggests that ACHD patients have a higher risk to develop CAD, acute myocardial infarction (AMI) and other acquired cardiovascular disease (ACVD) than their non-ACHD matches [[7], [8], [9]]. These data depict a worrisome picture. In fact, as CAD is known to be the most common aetiology of heart failure in non-ACHD patients [10], this growing load of CAD among young adult with CHD do sum up to the heart failure risk associated with the structural anomalies and the necessary cardiac surgeries that these patients undergo to correct or mitigate their congenital defects [11]. Thus, it is paramount for both congenital heart disease specialists and non-ACHD cardiologists to be aware of the cardiovascular risk of ACHD and make all efforts to prevent the development of CAD and other ACVD in this specific population. At the same time, they should be familiar with all particularities of CAD in ACHD.

### 1.1 Risk of coronary artery disease in ACHD

**Fig. 1:**
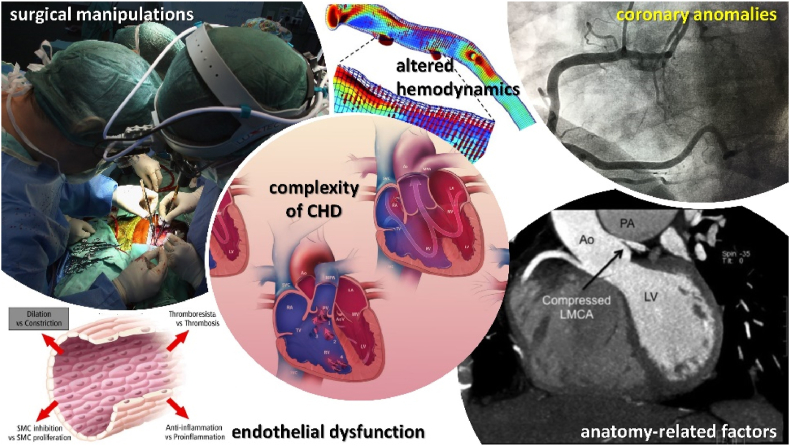
The figure depicts the main factors affecting the development of coronary artery disease (CAD) in adults with congenital heart disease (ACHD), including (clockwise starting on the upper left corner): surgical manipulation; altered hemodynamics; coronary anatomy anomalies; other anatomy-related factors (e.g. *ab estrinseco* compression of the LMCA); endothelial dysfunction; anatomic and clinical complexity of the congenital defect.

There has been a cogent debate whether CHD might per se contribute to increase the risk of CAD [12]. Predisposing CHD-related factors, such as coronary anomalies, surgical manipulations, and endothelial dysfunction, as well as conventional cardiovascular risk factors are likely to interact in specific congenital anomalies to cause CAD in ACHD [13,14]. Very recently, solid data from the Dutch nationwide registry suggested an increase in CAD risk in ACHD subjects. In fact, a substantial relative risk (RR) of CAD (RR = 12.0 in women; 4.6 in men) compared to the general population was registered. Results also showed an increase in CAD rates from 0.3 per 1000 patient-years at the age of 20 to 5.8 at the age of 70 in women and from 0.5 to 7.8 in men, demonstrating that this ACHD-related risk gap is larger in the female population. Also of note, the cumulative incidence of CAD was not significantly increased in mild CHD but was substantially increased by 2-fold in moderate and by 3-fold in severe CHD, suggesting that CAD risk increases with the severity of the congenital defect [8]. In line with these data, a recent gender-balanced cohort study involving 17189 ACHD subjects and 180131 age- and sex-matched controls without CHD uncovered a 1.6-fold higher risk for acute myocardial infarction. This risk difference was maintained even after correction for exposure to cardiovascular risk factors and was progressively amplified with the increase in patients’ age [15]. In the same study, ACHD patients also had a 1.4-fold higher risk of recurrent events after the index AMI [15]. These findings are in line with previous evidence involving 10501 CHD subjects, prospectively followed-up in a Danish nationwide cohort, showing a higher risk of AMI in ACHD compared to controls (Hazard Ratio = 2.0; 95% CI: 1.7–2.3) with a parallel increase in 30-day post-AMI mortality (Hazard Ratio = 1.4; 95% CI: 1.0–1.8). This increase in post-AMI mortality was higher in patients with severe CHD than in those with mild or moderate defects [5]. A graphical summary of the main factors influencing the development of CAD in ACHD is reported in Fig. 1.

What is the reason for the increased risk for ACVD among ACHD?

The role played by CHD-related aetiology on the increased risk in early adulthood is further supported by recent evidence showing that compared to controls from the general population, young CHD patients with ischemic heart disease from the Swedish registry had proportionately fewer traditional risk factors [9]. Along the same line, an English study including older ACHD patients showed a signal for increased CAD risk after adjusting for conventional cardiovascular risk factors, suggesting a contribution from CHD-related factors [6]. As people age, conventional risk factors may become more important in determining their risk of developing CAD: in an ACHD case-control study (mean age 55 years), CAD was linked to conventional (not CHD-related) risk factors [13]. Additionally, smoking [6], hypertension [6,16,17], diabetes [6,17,18], metabolic syndrome [19] are now more common, with 70–80% of ACHD patients having at least one conventional risk factor [16,20]. The higher prevalence of CHD-related risk factors in these patients, including anomalous coronary anatomy, may account for the greater risk-increase in relatively young patients with severe CHD. In fact, transposition of the great arteries is linked to (post-surgical) coronary abnormalities and an increased risk of developing coronary artery disease (CAD) [21], and other serious CHDs are linked to a high prevalence of coronary anomalies [22]. Premature CAD may be exacerbated by widespread endothelial dysfunction in cyanotic CHD [23]. Additionally, patients with severe CHD may have worse atherosclerotic risk profiles, as indicated by lower levels of physical activity [24] and a higher risk of developing diabetes [18]. The higher CAD risk increase in women with CHD cannot be explained by currently available data. The gender imbalance in prevalence of conventional cardiovascular risk in the general population (e.g. men tending to smoke more and having higher cholesterol and blood pressure levels) is usually less pronounced among ACHD subjects [16]. In addition, sex differences in traditional risk factors might not be attenuated if the risk is primarily influenced by CHD-related factors. This difference in risk between older ACHD men and the general population may be due to a selection bias caused by a higher early CHD-associated mortality in men with consequently a lower prevalence of CHD-associated risk factors in the elderly patients who survived [9]. Future research should examine the generalizability of these findings across different cohorts.

In addition, specific CHD are traditionally associated to a higher CAD risk. Among these, coarctation of the aorta (CoA) is strongly associated with CAD. In fact, despite no demonstration exists that CoA is an independent risk factor, CAD is the first cause of death among CoA patients [[25], [26], [27]]. Multiple hypotheses have been generated to explain this association. Among the others, one key element in these patients might be arterial hypertension. Notably, masked or uncontrolled hypertension was reported in almost half of children and adolescents after successfully treatment of CoA [28], and this picture is even larger in middle-aged CoA patients with a 75% prevalence of hypertension [29]. Another key risk element might be endothelial dysfunction, commonly found among CoA patients, including normotensive patients after correction [30]. A slightly more complex relationship links Turner syndrome to CAD, one of the most common death causes in these patients. In fact, on one hand the high prevalence of arterial hypertension also seems to play a role here [31], on the other hand, CoA is also common among Turner patients. Also, coronary anomalies are present in about 20% of women with Turner syndrome [32]. CAD is also very frequent in patients with transposition of the great arteries (TGA), where it was reported in 6.8–11.3% of patients after arterial switch operation (ASO), even though this relationship is often characterized by surgery-related mechanical obstruction [33,34], or more indirectly linked to the surgical manipulation of the proximal coronary segments, possibly causing endothelial damage and vascular dysfunction. Interruption of autonomous innervation of the coronary arteries during surgery also contributes to vascular dysfunction in TGA patients [35]. Noteworthy, for the same reason reported above, CAD might frequently go asymptomatic in TGA patents.

At the other end of the spectrum, patients with cyanotic heart defects might have some sort of protection against CAD [7,36]. However, many uncertainties are still source of debate about this effect. Among the mechanisms claimed to underly this phenomenon is the low prevalence of overweight, obesity and hypercholesterolaemia in patients with cyanosis [37]. In line with this hypothesis are the lower carotid intima media thickness (IMT) and the increased local availability of nitric oxide often reported in association with cyanosis [38]. Thrombocytopaenia was also suggested to be a CAD-fighting partner in cyanosis [36]. But all that glitters is not gold: in fact, CAD risk seems to catch up later in life, particularly after correction of the cyanogenic defect. As recently observed, coronary artery calcifications and other signs of subclinical CAD become common in middle aged patients with Eisenmenger syndrome [39]. In line with these findings, prevalence of CAD was reported to be significantly larger in older (>40 years-old) patients with tetralogy of Fallot (ToF) compared to controls [40,41].

### 1.2 Peculiarities of CAD in ACHD

Coronary artery disease (CAD) in patients with congenital heart disease (CHD) has several unique characteristics compared to CAD in the general population, as described in more details in Table 1. A recent large study involving a general population conducted in the Netherlands showed an increased risk for CAD within the ACHD subgroup [8]. In fact, the standardized Hazard Ratio (HR) of CAD was 1.3–2.9 compared to the general population. This incidence remained significantly higher until up to age of 65 years in women, and up to 50 years in men. On the other hand, slight differences were registered in similar studies run in Sweeden [9], Denmark [5] or the UK [6]. These differences might be partially explained through the different populations captured in the studies. In fact, young subjects from the Swedish registry had a proportionally lower risk attributable to classical cardiovascular risk factors, while the CHD-related risk might have played a stronger role in the English study, due to the older age of ACHD patients included. Prevalence of CAD was only slightly larger for more severe CHD in the Dutch study [8]: cumulative CAD incidence over 10 years was 1.4% in mild and 1.9% in moderate or severe CHD. On the contrary, the studies from Sweeden [9] and Denmark [5] documented a larger greater relative risk of CAD in more severe CHD, along with a larger relative risk in women [5].

**Table 1:** Key peculiarities of coronary artery disease in ACHD.

Specific characteristic	explanation
Increased prevalence	An increased prevalence of CAD has been reported in patients with CHD compared to the general population, particularly in those with repaired tetralogy of Fallot and other complex CHD.
Younger Age of Onset	The age of onset of CAD in patients with CHD is often younger compared to the general population, with some studies reporting that CAD can develop in patients with CHD as young as the third decade of life
Abnormal Anatomy	CHD can result in abnormal coronary artery anatomy or abnormal branching patterns, which can increase the risk of CAD.
Altered Hemodynamic	CHD can result in altered blood flow patterns, which can increase (or decrease in some cases) the risk of atherosclerosis and CAD
Surgical Interventions	Previous surgical interventions for CHD, especially if involving the manipulation of coronary arteries or their ostia, can increase the risk of CAD by altering the normal blood flow in the coronary arteries
Concomitant Risk Factors	Adult patients with CHD often have multiple concomitant risk factors for CAD, such as hyperlipidaemia, diabetes, and hypertension, which can exacerbate the risk of CAD
Diagnosis and Management	The diagnosis and management of CAD in patients with CHD can be challenging due to the complex anatomy and altered hemodynamic associated with CHD. However, the use of imaging techniques, such as coronary angiography, and the implementation of appropriate preventive measures and treatment strategies can help reduce the risk of CAD in these patients

Early onset CAD is more frequent among ACHD subjects than in the general population [8]. ACHD patients also tend to be younger at the onset of the first AMI [15]. On the contrary, the periprocedural myocardial infarction risk after cardiac surgery was comparable between the groups [15]. In line with these findings, colleagues from the cardiovascular surgery of the Mayo Clinic recently reviewed their historical cohort of ACHD patients undergoing Coronary Artery Bypass Surgery (CABG) during the surgical treatment of the congenital defect [42]. They report progressive reduction of perioperative mortality from the 1970s to our days, to similar rates measured in non-ACHD CABG surgery [42].

As commonly happens with non-CHD patients the frequent overlap between acute coronary syndromes (ACS) and myocardial injury related to hemodynamic overload or imbalance due to multiple factors can be challenging when assessing ACHD subject with suspected ACS in the acute setting, especially when a single snapshot is available, especially since high-sensitivity troponin assays became available [[43], [44], [45], [46]]. In fact, the hemodynamic overload associated to the excess volume in the right-heart circulation in patients with atrial septal defects (ASD), ventricular septal defects (VSD), or in general with left-to-right shunt can substantially impact on blood levels of cardiac necrosis biomarkers [47].

In line with these observations, a recent study reported that among patients presenting to the emergency department (ED) with chest pain, a confirmed diagnosis of AMI was less common among ACHD patients (5.2%) compared to controls (19.7%), in favour of alternative diagnoses being more common in the ACHD group, such as arrhythmias (14% vs 6%; p < 0.001) and acute heart failure (3% vs 0.3%; p = 0.02). This poses a relevant issue for the initial management of ACHD patients in the emergency setting, which requires healthcare personnel dealing with chest pain in the ED to be aware of the different epidemiology of ACHD subjects [48]. In addition, use of score system to better stratify CAD risk, especially among ACHD should be encouraged. Furthermore, the development of ACHD-specific atherosclerotic risk stratification systems should be pursued on a longer term. In this regard, the use of advanced computational models might be of help in optimize and personalize the clinical management of these patients [49].

### 1.3 Diagnosis and treatment of CAD in ACHD

**Fig. 2:**
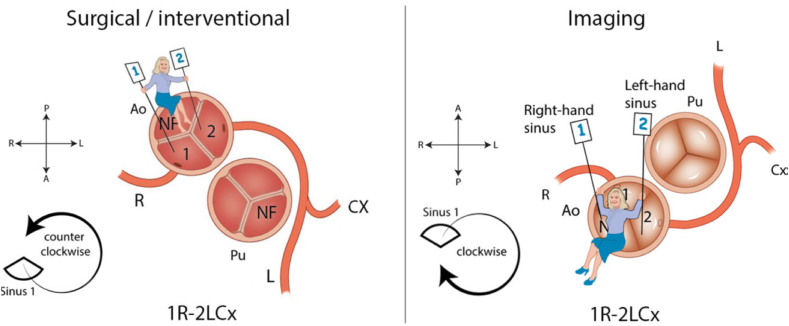
The figure depicts the Leiden Convention for coronary coding, in the standard *surgical* view (**left panel**) and the *universal/imaging* view (**right panel**). Reprinted from The Journal of Thoracic and Cardiovascular Surgery, 156/6:6–10, Adriana C. Gittenberger-de Groot, Wilke M.C.Koenraadt, Margot M. Bartelings, Regina Bökenkamp,MarcoC. DeRuiter, Mark G. Hazekamp,Ad J.J. C. Bogers,Jan M.Quaegebeur, Martin J. Schalij, Hubert W. Vliegen, Robert E.Poelmann, Monique R.M. Jongbloed. Coding of coronary arterial origin and branching incongenital heart disease: The modified Leiden Convention, Copyright (2018), with permission from Elsevier (License Number: 5490310144912).

Coronary angiography and angioplasty present unique challenges in patients with congenital heart disease (CHD), for multiple reasons, starting from the vascular access, that should be carefully selected depending on specific patients' anatomy or previous surgical procedures. The prevalence of coronary artery anomalies is increased from 0.3 to 1.3% in subjects with normal cardiac anatomy to 3–36% in ACHD patients [[50], [51], [52], [53], [54]]. Recently, the use of computed tomography angiography (CTA) imaging to characterize coronary anomalies is emerging as a useful tool. In fact, on one hand it is a practical diagnostic aid to minimize the recourse to invasive imaging. On the other hand, it can be very useful in specific cases, such as the fine anatomical characterization of anomalous coronary arteries originating from the opposite sinus of Valsalva (ACAOS), where CTA can easily identify anatomic markers of high clinical risk, such as a slit-like ostium, narrow-angle take-off, proximal luminal narrowing, an intramural segment and even measure the distance between the lumen of the aorta and the lumen of the coronary artery [55]. ACHD patients often have unique and complex anatomy or coexisting cardiac lesions that can make the selective cannulation of the coronary arteries more difficult. Similarly, interpretation of coronary angiography can be more complex, as it is required to know how to recognize and assess specific anomalies, such as those resulting from ab extrinsic compression, for example in the case of pulmonary hypertension or abnormal anatomical and/or post-surgical structures [[56], [57], [58]]. Along the same line, complementing imaging techniques might be required in ACHD patients with challenging imaging. Furthermore, the risk of periprocedural complications, such as bleeding, contrast-induced kidney injury, arrhythmias or hemodynamic instability may be increased in ACHD patients. For all these reasons, it is important for teams performing coronary procedures to be familiar with the specific anatomy, surgical history and complexities of CHD and to have access to subspecialty expertise in CHD. In this context, it is paramount to develop a dedicated workflow to perform coronary procedures in ACHD patients, paying careful attention to all steps of the workflow. Table 2 summarizes step-by-step tips to performing coronary procedures in ACHD patients. Even though a multidisciplinary approach is always desirable when dealing with ACHD, general knowledge about CHD among adult cardiologists is critical to better contextualize the patients' clinical picture and to ensure a safe and effective invasive management. In fact, specific congenital defects are usually associated to well-known coronary alterations, such as coronary anomalies, compression by abnormal vascular structures, rotation of the aortic root, as described in a more detailed way in Table 3. At the same time, it is important to consider that each patient with ACHD is unique and may require a tailored approach [59]. Approaching patients with known coronary anomalies, it can be very helpful to be familiar with the Leiden Convention for coronary coding. Originally only adopted by surgeons, a modified, universal Leiden Convention has been developed to allow wider use by imaging specialists dealing with CT- or MR-imaging, as well as to interventional cardiologists and clinicians [60,61]. It's not a classification *stricto sensu*, but a simple coding system that is broadly applicable and quickly provides details on the anatomical configuration of coronary origin from the aorta. Briefly, the coding system uses a combination of letters and numbers to describe the anatomical configuration of coronary arteries, where the letter refers to the specific coronary artery (L for the left anterior descending; Cx for the circumflex artery and R for the right coronary artery), while the number refers to the specific coronary sinus. Origin of each coronary artery from a specific sinus of Valsalva is described counterclockwise in the surgical view and clockwise in the universal view (Fig. 2). By providing a standardized and reproducible method of describing coronary anatomy, it helps to improve multidisciplinary communication.

**Table 2:** Step-by-step tips for coronary artery procedures in ACHD.

Steps	Tips
Pre-procedure Planning	Pre-procedural planning (including a detailed patient's history, surgical history, imaging) is extremely important for a safe and successful coronary procedure in ACHD. Collaboration with a CHD specialist can be very helpful
Vascular Access	Confirm patency of the subclavian arteries prior to undertake a radial access, especially in patients might have a classic Blalock-Taussig shunt (older patients with tetralogy of Fallot, pulmonary atresia, right ventricular hypoplasia)
Imaging integration	Cutting-edge multi-modality imaging (including computed tomography, magnetic resonance, advanced echocardiography) should be exploited to obtain the most detailed anatomical picture of the patient prior to the invasive procedure
Catheter Selection	Appropriate size and type of intravascular devices are a key to minimize procedural risks and to improve procedural quality
Injection Technique	Optimization of the injection technique is very important: a slow, controlled injection can be helpful to obtain good-quality images, reduce vascular complications and minimize the risk for contrast-induced nephropathy
Team Approach	A multidisciplinary team, including interventional cardiologists, congenital heart disease specialists and expert image specialists is very helpful, particularly in more complex cases
Close Monitoring	Real time clinical monitoring during the invasive procedure is mandatory for safety and to ensure patient's well-being

**Table 3:** Coronary artery features frequently associated with specific congenital defects.

Specific congenital condition	Issue	Suggestion
Tetralogy of Fallot (ToF)	aortic root is usually dilated and twisted clockwise [59]	ostium of the right coronary artery is more anterior than usual
Tetralogy of Fallot	coronary anomalies can be present: a coronary artery crossing the right ventricular outflow tract is common [62,63]	computed tomography angiography (CTA) is useful to assess the abnormal course, frequently associated with myocardial bridge
ToF and other cono-truncal anomalies	Risk of iatrogenic compression during percutaneous pulmonary valve (PV) intervention	Assess coronary artery course before planning PV interventions
Transposition of the great arteries	ventriculoarterial discordance is frequently associated with coronary artery anomalies, making both diagnosis and treatment more challenging	careful evaluation of coronary anatomy is key, e.g. using cardiac magnetic resonance imaging; adoption of both pre-procedural (stress-perfusion imaging) and intra-procedural functional testing (FFR/iFR) can be helpful
Congenitally corrected transposition of the great arteries	the left coronary artery usually arises from the right coronary sinus of Valsalva (and irrorates the right-sided sub-pulmonary ventricle), while the right coronary artery arises from the left sinus of Valsalva (irrorating the hypertrophied systemic right ventricle)	cannulation of the left main with the right Judkins (JR) and of the right coronary using the left Judkins (JL) catheters
Pulmonary arterial hypertension or secondary pulmonary hypertension due to aortopulmonary window or Eisenmenger syndrome, after placement of a prosthetic pulmonary valve or a stent in a PA conduit	*ab-extrinseco* compression of the left main coronary artery (LMCA) [64,65]	CTA or coronary angiography can easily confirm the suspect. LMCA stenting is associated to low mortality and symptom relief [58,66,67]

Thorough anatomic and functional assessment is a critical issue in ACHD patients. The combination of multiple diagnostic modalities, including echocardiography, cardiac catheterization, CT- and MR-imaging can provide a better guide to patient management [68]. However, their results should be carefully evaluated in women, as negative functional tests have a poor diagnostic performance, especially for coronary anomalies of the RCA [69,70]. In these cases, intracoronary imaging [70,71] or intracoronary pressure measurements can be a valuable option [72,73]. However, both invasively measured and non-invasive CT-derived coronary pressure indices should be carefully interpreted. A recent study reported a moderate reduction of FFR-CT values in coronary arteries with congenital anomalies, however this was associated with a low risk of adverse cardiovascular events at 5 years [74]. Nevertheless, FFR-CT values are able to improve the capacity of CTA to identify high-risk anatomical features in these patients [75]. In specific cases, such as coronary anomalies with inter-arterial course [76,77] additional pharmacological testing (e.g. with dobutamine administration) may be required for functional assessment [70,71]. In those cases where coronary pressure measurement should be performed during provocative tests, use of adenosine-free coronary pressure indices can offer practical advantages.

The recent further developments of MR-imaging, particularly with the possibility to perform a MR-based coronary angiography (MRCA), offers the chance to assess coronary anatomy in an accurate and reliable 3D modality, overcoming most limitations presented by 2D techniques, such as x-ray angiography or 2D-echocardiography [[78], [79], [80]]. Furthermore, MR-imaging can provide useful information on cardiac anatomy and function, both at rest and under pharmacological stress [81,82]. In addition, in contrast to X-ray angiography and CT-imaging, it has the advantage to avoid further exposition to ionizing radiations in subjects that typically undergo frequent imaging such as ACHD patients.

The recent advancements of computed tomography (CT), characterized by a substantial increase in speed of acquisition, lower total dose and higher temporal and spatial resolutions brought along an improvement in the assessment of coronary arteries and of the heart are valuable for the management of ACHD [22]. These advancements translate in better visualization of moving structures with less motion artifact, allowing for a more reliable assessment of the relationship of coronary arteries with adjacent anatomical structures [83].

Percutaneous coronary interventions (PCI) can present specific challenges in ACHD patients, due to abnormal cardiac or vascular anatomy or to previous corrective surgery. For this reason, knowledge of the specific congenital anomaly, patient's anatomy and surgical history is crucial before undertaking coronary interventions in these patients. This can sometimes be an issue in the context of emergent procedures such as in ACS. Although rare, performing a PCI in dextrocardia is a case in point [[84], [85], [86], [87], [88], [89], [90], [91], [92]]. The main challenge is represented by the unfamiliar image orientation, making cannulation of coronary arteries but also the managing of the entire procedure cumbersome, with potential increase in fluoroscopic time, radiation dose and contrast medium amount [84,88]. In fact, catheter orientation to meet the ostia, catheter rotation for cannulation of the right coronary artery (RCA) and wire orientation all work the other way around. In this situation, a very simple tool can be very practical: modulation of the angiographic view using the horizontal sweep reverse function – a feature available in most catheterization facilities - returns a view that resembles the usual angiography [86,90].

Usually quite rare among CAD patients, extrinsic compression of coronary vessels is not a rarity among ACHD. Compression of the Left Main Coronary Artery (LMCA) can be found in association with an enlargement of the pulmonary artery (PA) [93], pulmonary hypertension [58,66], transcatheter implantation of pulmonary valve prosthesis [94], the placement of a stent in a PA conduit [95], an unruptured left sinus of Valsalva aneurysm (SVA) [96,97], a ventricular pseudoaneurysm [98], among the others conditions related to CHDs. Despite the LMCA being the most frequently coronary segment subject to extrinsic compression, similar stenoses are also reported on the left anterior descending artery (LAD) or the right coronary artery (RCA) due to SVA [96,99]. In addition, symptomatic dynamic compression of the LMCA by the PA is not infrequent in patients with pulmonary arterial hypertension (PAH), with symptoms resembling acute coronary syndromes [93]. Confirmation of the nature of the lesion and its hemodynamic relevance, often requires an integrated approach using multimodality imaging using both non-invasive and invasive imaging [58]. This is particularly true in case of dynamic obstructions, where CT imaging can provide precise anatomic characterization and indirect markers of dynamic compression, such as the distance between the lumen of the intramural coronary segment and the pulmonary artery, while the simultaneous injection of contrast dye during invasive angiography to assess the spatial interaction between the coronary lumen and the compressing structure (e.g. the pulmonary artery, an SVA, a post-surgical ventricular aneurism) can be very useful in understanding the actual clinical relevance of the lesion. A recent meta-analysis summarizing available evidence showed that a simple index such as PA diameter is a predictor of LMCA compression [66]. The most frequently adopted treatment is percutaneous coronary intervention (PCI) with stent implantation, followed by coronary artery bypass graft (CABG) and PA interventions. PCI is effective for angina relief and is associated with low mortality during the follow up [66].

Being exposed to surgical procedures for primary and secondary correction of congenital defects, a certain number of ACHD undergo concomitant CABG in case of relevant atherosclerotic coronary artery disease, as described in a report by the Mayo Clinic, portraying an ACHD cohort with a median age of 64 years at surgery [100]. The CABG procedure consisted in a single bypass in 57% of patients, while 2 bypasses were necessary in 26%. A left internal mammary artery (LIMA) to the LAD bypass was used in 70% of cases. Of note, a LIMA-to-LAD bypass could not be used in some cases due to previous crossbow incision thoracotomy [100].

### 1.4 Prognosis of CAD in ACHD

Among ACHD subjects undergoing coronary revascularization by means of CABG during surgical correction of a congenital cardiac defect for atherosclerotic CAD long-term survival progressively dropped from 91% at 1 year, 76% at 5 years, 56% at 10 years, and 33% after 15 years [100]. Noteworthy, while male patients undergoing CABG in combination with CHD repair had similar long-term survival compared with controls, women had significantly lower survival rates at 5 and 10 years compared with controls [100].

In addition to the higher risk of developing AMI, recurrent AMI and consequently newly diagnosed heart failure, which contributed to their increased risk of developing MI, recurrent MI, new-onset heart failure, ACHD patients tend to have a worse prognosis after an AMI [15]. In fact, assessing the risk of myocardial infarction (MI) and the long-term outcomes after MI in a cohort of 17189 middle-aged and older patients with congenital heart disease (ACHD) aside 180131 sex- and age-matched controls, Fedchenko M. et al. found that ACHD had a 1.4 times increased risk of the composite of recurrent MI, new-onset heart failure, or death after the index MI, compared to controls. To date, no evidence is available to explain the negative prognostic impact of congenital defects on acquired CAD and we cannot exclude that volume overload, previous or ongoing cyanosis, residual ischemia or previous cardiac surgeries may play a role. At the same time, the increased complexity of diagnostic and therapeutic interventions might also be a partaker, especially in more complex anatomies. Noteworthy, this result was mainly due to the higher incidence of newly diagnosed heart failure in patients with ACHD (42.2%) compared to controls (29.5%) [15]. Again, no solid knowledge is available to understand the underlying mechanisms. However, it is tempting to hypothesize that residual haemodynamic impairment related to previous surgical corrections or to residual ischemic myocardial scar are partners in crime [101].

Hence, ACHD patients suffering for an AMI not only have a worse prognosis compared to non-ACHD controls, but this effect seems to be larger with more complex congenital defects [5]. For these reasons, early identification of cardiovascular risk factors, their optimal management as well as prompt diagnose of acute coronary events are of the utmost importance to minimize morbidity and mortality associated to AMI.

## 2 Conclusions

Altogether, the available clinical evidence points at an increased CAD risk in ACHD subjects, with a more pronounced risk difference at younger age, but also in women and in patients with more complex/severe CHD. While ongoing and future research will shed new light on the mechanisms underlying the higher-than-expected CAD risk among ACHD subjects, the current knowledge strongly supports the importance of careful screening for CAD and the early management of known cardiovascular risk factors in these patients.

## Declaration of competing interest

The authors declare that they have no known competing financial interests or personal relationships that could have appeared to influence the work reported in this paper.
